# On *Rickettsia* Nomenclature

**DOI:** 10.3201/eid14.3.080065

**Published:** 2008-03

**Authors:** Robert F. Massung, William L. Nicholson, Marina E. Eremeeva, Gregory A. Dasch

**Affiliations:** * Centers for Disease Control and Prevention, Atlanta, Georgia, USA

**Keywords:** rickettsia, bacterial nomenclature, taxonomy, commentary

## Abstract

On *Rickettsia* Nomenclature

This issue of Emerging Infectious Diseases contains 2 independent reports of *Rickettsia sibirica* infections in Spain and Portugal. The authors identify the agent both as a subspecies (*1*) and as a strain (*2*). This inconsistency reflects a lack of consensus regarding the use of subspecies designations for *Rickettsia* taxa, so appropriate designation of these pathogens as strains or subspecies remains problematic.

Tick-borne typhus of northern Asia (or North Asian spotted fever) was first discovered in the 1930s, and its etiologic agent, *R. sibirica,* was formally described in 1949. In 1993, an isolate designated “*mongolitimonae*” was first discovered in the People’s Republic of China (*3*), considered a new species (*4*), and finally described as a new genotype of *R. sibirica* (*5*). A proposal to create 2 subspecies, *R. sibirica sibirica* and *R. sibirica mongolitimonae*, was recently published (*6*). A new prokaryote name must be both effectively and validly published. To become effectively published, a name must meet certain rules, as defined by the International Code of Nomenclature of Bacteria (*7*). To become validly published, the name must then appear on a Validation List published in the International Journal of Systematic and Evolutionary Microbiology (*8*). This provides an orderly system for bacterial names to be properly introduced and published in the scientific literature. Valid and nonvalid names are listed on regularly updated websites (*9*,*10*). Names introduced without being validated have no standing in bacterial nomenclature. That is currently the status of these proposed *R. sibirica* subspecies. Therefore, the use of strain designations is still appropriate.

If, and when, the subspecies names are validated, they are likely to be adopted and routinely used in the literature. Rickettsial taxonomy continues to evolve, and future changes should be determined by critical scientific judgments and general consensus within the scientific community.

Dr Massung is chief of the Rickettsial Zoonoses Branch, Division of Viral and Rickettsial Diseases, National Center for Zoonotic, Vector-borne and Enteric Diseases, Centers for Disease Control and Prevention. His research interests include laboratory and epidemiologic investigations targeting the detection, prevention, and control of rickettsial and *Bartonella* spp. diseases and Q fever.

## References

[1] Aguirrebengoa K, Portillo A, Santibáñez S, Marín JJ, Montejo M, Oteo JA. Human *Rickettsia sibirica mongolitimonae* infection, Spain. Emerg Infect Dis. 2008;14:528–9 18494099 10.3201/eid1403.070987 PMC2570842

[2] de Sousa R, Duque L, Poças J, Torgal J, Bacellar F, Olano JP, Portuguese patient infected with *Rickettsia sibirica.* Emerg Infect Dis. 2008;14:529–31 18325289 10.3201/eid1403.070680 PMC2570837

[3] Yu X, Jin Y, Fan M, Xu G, Liu Q, Raoult D. Genotypic and antigenic identification of two new strains of spotted fever group rickettsiae isolated from China. J Clin Microbiol. 1993;31:83–8.[REMOVED HYPERLINK FIELD] 8093253 10.1128/jcm.31.1.83-88.1993 PMC262626

[4] Fournier PE, Tissot-Dupont H, Gallais H, Raoult DR. *Rickettsia mongolotimonae*: a rare pathogen in France. Emerg Infect Dis. 2000;6:290–2. 10827119 10.3201/eid0603.000309 PMC2640873

[5] Fournier PE, Dumler JS, Greub G, Zhang J, Wu Y, Raoult D. Gene sequence-based criteria for identification of new *Rickettsia* isolates and description of *Rickettsia heilongjiangensis* sp. nov. J Clin Microbiol. 2003;41:5456–65. 10.1128/JCM.41.12.5456-5465.2003 14662925 PMC308961

[6] Fournier PE, Zhu Y, Yu X, Raoult D. Proposal to create subspecies of *Rickettsia sibirica* and an emended description of *Rickettsia sibirica.* Ann N Y Acad Sci. 2006;1078:597–606.[REMOVED HYPERLINK FIELD] 10.1196/annals.1374.120 17114787

[7] Lapage SP, Sneath PHA, Lessel F, Skerman VBD, Seeliger HPR, Clark WA, editors. International code of nomenclature of bacteria (1990 revision). Bacteriological code. Washington: American Society for Microbiology; 1992. 21089234

[8] Tindall BJ, Kämpfer P, Euzéby JP, Oren A. Valid publication of names of prokaryotes according to the rules of nomenclature: past history and current practice. Int J Syst Evol Microbiol. 2006;56:2715–20. 10.1099/ijs.0.64780-0 17082418

[9] Euzéby JP. List of prokaryotic names with standing in nomenclature. 2007. [cited 2008 Jan 2]. Available from http://www.bacterio.cict.fr

[10] Euzéby JP. List of prokaryotic names without standing in nomenclature. 2007 Dec 29 [cited 2008 Jan 2]. Available from http://www.bacterio.cict.fr/nonvalid.html

